# Impact of metformin on C-reactive protein levels in women with polycystic ovary syndrome: a meta-analysis

**DOI:** 10.18632/oncotarget.16019

**Published:** 2017-03-08

**Authors:** Yong Chen, Meng Li, Hongli Deng, Sheying Wang, Lihua Chen, Ningsha Li, Dan Xu, Qiguang Wang

**Affiliations:** ^1^ Department of Clinical Laboratory, The First Hospital of Changsha City, Hunan, People’s Republic of China; ^2^ Department of Clinical Laboratory, The First Affiliated Hospital of Guangxi Medical University, Guangxi, People’s Republic of China; ^3^ Department of Clinical Laboratory, People’s Hospital of Liuyang City, Hunan, People’s Republic of China; ^4^ Department of Clinical Laboratory, The First Affiliated Hospital of Hunan Normal University, People’s Hospital of Hunan, Hunan, People’s Republic of China

**Keywords:** metformin, CRP, polycystic ovary syndrome, meta-analysis

## Abstract

The impact of the recommended first-line treatment with metformin on C-reactive protein (CRP) levels in patients with polycystic ovary syndrome (PCOS) is still controversial. We conducted a meta-analysis of studies reporting the impact of metformin on serum CRP levels in women with PCOS. The weighted mean differences (WMDs) and the corresponding 95% confidence intervals (CIs) were used to assesse the effects. GRADE approach was used to assesse the quality of the evidence. A total of 20 studies that included 433 women with PCOS were analyzed. CRP levels significantly decreased after metformin treatment (WMD = -1.23mg/L, 95%CI: -1.65 to -0.81, *I*2 = 93% and *P* < 0.001 for heterogeneity). The decreased levels of CRP were observed both in lean (BMI<25 kg/m^2^) and obese (BMI>25 kg/m^2^) patients. Interestingly, the degree of decreased CRP levels was not depended on metformin dosage, but more significantly in patients treated beyond 6 months (WMD_≥6months_ = -1.47mg/L *vs*. WMD_<6months_ = -0.94 mg/L). Decreased CRP levels are not associated with the status of IR and androgen in patients with PCOS. However, the quality of evidence was very low because of the limitations and inconsistency of the included studies. Therefore, metformin shows the potential effects on CRP levels in women with PCOS. However, considering the very low quality of evidence for the analysis, the effect of metformin on CRP levels are still very uncertain, and large-scale randomized-controlled study is needed to ascertain the long-term effects of metformin in PCOS.

## INTRODUCTION

Polycystic ovary syndrome (PCOS) is a common endocrinopathy with various clinical features. According to criteria from Rotterdam [1], the National Institute of Health (NIH) [2], and the Androgen Excess and PCOS Society in 2006 [3], the key characteristics features of PCOS are clinical or biochemical hyperandrogenism, menstrual irregularity, and an ultrasound picture of PCO. Although obesity and insulin resistance (IR) are not included in these diagnostic criteria, 61%-76% of women with PCOS are overweight [4], and IR is seen in 95% of obese and 65% of lean women with PCOS [5]. Therefore, an association between PCOS and the long-term consequences, such as type 2 diabetes (T2DM) and cardiovascular diseases (CVD), has been recognized [6].

PCOS is related to low-grade chronic inflammation [6, 7]. The C-reactive protein (CRP), an acute-phase protein produced mainly by hepatocytes, plays an important role in low-grade chronic inflammation and oxidative stress, especially in women with CVD [8]. Elevated CRP levels are also positively associated with IR and the incidence of T2DM [9]. Hence, high CRP levels are considered a potential cause of the long-term consequences of PCOS [10]. Indeed, Toulis *et al.* [11] demonstrated that serum CRP concentrations were significantly elevated in women with PCOS compared with controls. In multiple regression analyses, elevated levels of CPR are independently predicted by higher body fat and lower insulin sensitivity in women with PCOS. Therefore, high CRP levels may be an intrinsic characteristic of PCOS, and efforts to prevent or to decrease the serum CRP levels could benefit patients with PCOS.

Metformin, an oral anti-diabetic biguanide drug, could not only benefit hyperinsulinemia, but could also decrease the total and free testosterone levels in patients with PCOS [12-14]. Therefore, metformin is suggested as the first-line treatment for PCOS [6]. Recent preclinical and clinical studies have suggested that metformin has a direct anti-inflammatory action [15]. Considering the relationship between CRP levels and chronic inflammation, IR, the beneficial effects of metformin treatment on CRP levels in patients with PCOS could be expected. However, previous studies about this possible association have yielded contradictory results. Some studies observed significantly decreased CRP levels in patients with PCOS after metformin treatment [12, 16-24], but these results were not confirmed by other studies [25-29]. The relatively small sample sizes of most of these studies could be to blame, at least in part, for the inconsistent results. Furthermore, the impact of metformin dose and treatment duration on CRP levels is unclear. We therefore conducted a meta-analysis to quantify the impact of treatment with metformin on CRP levels in patients with PCOS.

## RESULTS

### Characteristics of the eligible studies

**Figure 1:**
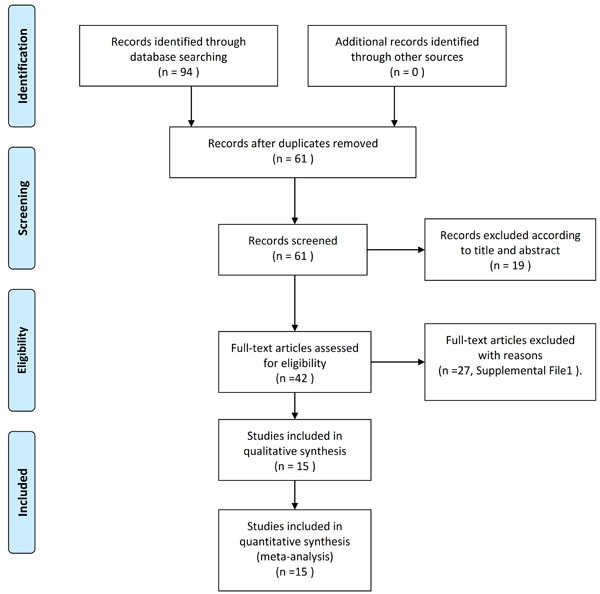
Flow diagram of literature review

Figure 1 provides an outline of the selected studies. Fifteen articles [12, 16-29] representing 433 participating women with PCOS were identified as suitable literature for this meta-analysis. The study by Behradmanesh *et al*. [25] was separated into three studies because they included different BMIs and sample sizes. The study by Morin-Papunen *et al*. [22] was separated into four studies, as they included different BMIs and metformin treatment duration and dose levels. Therefore, a total of 20 studies were pooled into this meta-analysis. The Rotterdam criteria were used in most of the included studies [12, 16-20, 23, 24, 26-29]; the Homburg criteria were used in two articles [22, 25], and only one study used the NIH diagnostic criteria [21]. Small sample sizes ( < 10) were found in three articles (including six studies) [21, 22, 28]. All the studies used mg/L as the unit for CRP, and immunoturbidimetry method was used in most of studies (12 of 20) to measure serum CRP levels. Three articles [22, 24, 28] (including 7 studies) were assessed as “Low” quality and the other studies were all “High” quality according to the quality score assessment. The main characteristics of the included studies are provided in Table 2.

**Table 1:** Scale for quality assessment

**Criteria**	**Score**
**1. Is the case definition adequate?**	
Yes, clearly defined objective according to criteria of diagnosis of PCOS	2
No criteria of diagnosis, but with independent validation	1
No, or no description	0
**2. Is the exclusion criteria described adequate?**	
Yes. clearly described	2
With potential selection biases (e.g., other diseases or pregnancy)	1
No, or no description	0
**3. Confound factors**	
Baseline characteristics clearly described (e.g., BMI, hirsutism)	2
No, or no description	0
**4. Dosage and usage of metformin**	
Clearly described	2
No, or no description	0
5. Sample sizes	
≥50	2
>10,<50	1
≤10	0

**Table 2:** The characteristics of the included studies in meta-analysis

										**CRP (mean±SD.mg/L)**				
**Study**	**Year**	**Country**	**PCOS Definition**	**No**.	**BMI**	**Age**	**Time** **(months)**	**Dose(mg/day)**	**Method**	**pre**	**post**	**HOMA-IR ratio**	**T ratio**	**Score**
Banaszewska	2009	USA	Rotterdam	36	<25	25.2±0.7	3	1700	ECLIA	2.56±0.64	1.23±0.7	0.90	0.86	8
Banaszewska	2011	USA	Rotterdam	47	<25	26.0 ±0.6	6	1700	ECLIA	2.5±0.5	1.18±0.7	1.05	0.82	8
Behradmanesh	2011	Iran	Homburg	12	<25	22.5±4.5	3	1500	IRMA	1.4±0.73	1.3±0.4	0.62	NR	9
Behradmanesh	2011	Iran	Homburg	14	≥25	22.5±4.5	3	1500	IRMA	5.07±5.7	2.9±1.9	1.21	NR	9
Behradmanesh	2011	Iran	Homburg	19	≥25	22.5±4.5	3	1500	IRMA	2.8±2.4	2.5±2.8	1.39	NR	9
Celik	2012	Turkey	Rotterdam	20	≥25	25.9±5.7	3	2000	ITM	0.72±0.4	0.3±0.2	0.84	0.91	8
Cetinkalp	2009	Turkey	Rotterdam	47	≥25	NR	4	2000	ITM	1±1.86	0.28±0.3	0.86	0.85	8
Diamanti-Kandarakis	2006	Greek	Rotterdam	22	≥25	24.3± 0.6	6	1700	ELISA	1.92±0.6	0.52±0.3	0.67	0.89	8
Esfahanian	2013	Iran	Rotterdam	17	≥25	21.9±9.3	3	2000	ITM	5.2±2.5	3.7±1.9	0.80	0.63	8
Heutling	2008	UK	Rotterdam	21	≥25	27.8± 4.7	6	1700	ITM	4±3	4±2.2	0.84	0.70	8
Hoeger	2008	USA	Rotterdam	6	≥25	16± 1.7	6	2000	CLIA	3.6±2.7	2.8±2	0.88	1.04	5
Jakubowska	2008	Poland	Rotterdam	29	≥25	28.2±6.3	6	1000	ITM	3.53±3.64	3.73±3.7	0.98	0.59	9
Mohiyiddeen	2013	UK	Rotterdam	17	≥25	30.0± 0.9	3	1000	CLIA	4.11±1.09	1.98±0.6	0.66	0.83	9
Mohlig	2004	Germany	NIH	9	≥25	28.9±0.7	6	2550	ITM	3.34±0.82	1.92±0.3	1.20	0.71	6
Morin-Papunen	2003	Finland	Homburg	8	<25	28.2±1.2	3	1000	ITM	1.34±0.39	1.2±0.4	NR	NR	5
Morin-Papunen	2003	Finland	Homburg	8	≥25	29.5±1.1	3	1000	ITM	4.83±1.05	2.85±0.9	NR	NR	5
Morin-Papunen	2003	Finland	Homburg	8	<25	28.2±1.2	6	*1000-2000	ITM	1.34±0.39	0.92±0.3	NR	NR	5
Morin-Papunen	2003	Finland	Homburg	8	≥25	29.5±1.1	6	*1000-2000	ITM	4.83±1.05	2.11±0.4	NR	NR	5
Orio	2007	Italy	Rotterdam	50	≥25	28.5±3.1	6	1700	ITM	1.8±0.9	1.1±0.6	0.54	0.93	10
Velija-Ašimi	2007	Bosnia and Herzegovina	Rotterdam	35	≥25	20-35	12	850	ITM	6.37±1.72	1.67±0.7	NR	0.72	7

Abbreviations: CRP, C-reactive protein; HOMA-IR, homeostasis model assessment of insulin resistance; T ratio: total testosterone ratio; CLIA, Chemiluminescent immunoassay; ITM, immunoturbidimetry; IRMA, immunoradiometric assay; ECLIA, electrochemiluminescence immunoassay; ELISA, Enzyme-linked immunosorbent assay; SD, standard deviation; NR, not reported;*1000-2000, 1000mg/day for 3 months, then 2000 mg/day for 3 months. Score, quality score

### Meta-analysis results

**Figure 2:**
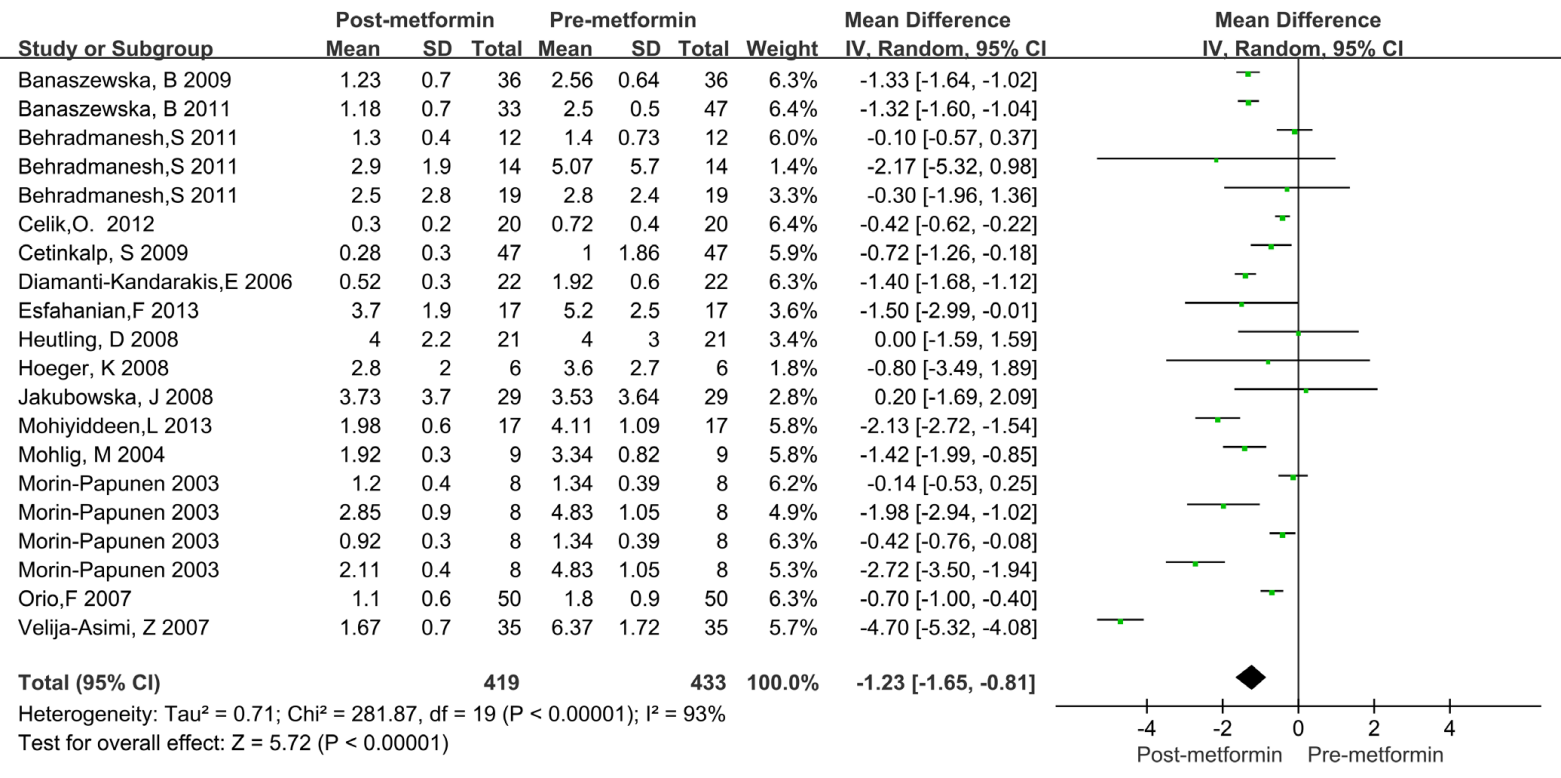
Forest plot for pooled quantitative synthesis

As shown in Figure 2 and Table 3, significant heterogeneity was observed in the pooled analysis (*I*2 = 93% and *P*Q < 0.001); therefore, a random-effects model was used, and the pooled results indicated that CRP levels significantly decreased after metformin treatment in women with PCOS (WMD = -1.23mg/L, 95%CI: -1.65 to -0.81, *P* < 0.001).

**Table 3:** Subgroup analysis results in meta-analysis

				**Heterogeneity**		**Quality of the evidence(GRADE)**
**Subgroup**	**No. study**	**WMD(95%CI)**	***P*** **value**	***I*^2^ (%)**	***P***	
Overall	20	-1.23 [-1.65, -0.81]	<0.001	93	<0.001	⨁◯◯◯^1,2^ VERY LOW
BMI						
<25	6	-0.79 [-1.28, -0.30]	<0.001	91	<0.001	⨁◯◯◯^1,2^ VERY LOW
≥25	14	-1.48 [-2.16, -0.80]	<0.001	94	<0.001	⨁◯◯◯^1,2^ VERY LOW
Duration(months)						
<6	10	-0.94 [-1.41, -0.47]	<0.001	87	<0.001	⨁◯◯◯^1,2^ VERY LOW
≥6	10	-1.47 [-2.16, -0.78]	<0.001	95	<0.001	⨁◯◯◯^1,2^ VERY LOW
Dose(mg/day)						
<2000	14	-1.21 [-1.32, -1.09]	<0.001	94	<0.001	⨁◯◯◯^1,2^ VERY LOW
≥2000	6	-0.55 [-0.71, -0.40]	<0.001	86	<0.001	⨁◯◯◯^1,2^ VERY LOW
HOMA-IR ratio						
<0.735	4	-1.07 [-1.76, -0.38]	<0.001	93	<0.001	⨁◯◯◯^1,2^ VERY LOW
0.735-0.865	4	-0.49 [-0.72, -0.25]	<0.001	7	0.360	⨁◯◯◯^2^ VERY LOW
0.865-1.015	3	-1.06 [-1.86, -0.26]	<0.001	23	0.270	⨁◯◯◯^2^ VERY LOW
<1.015	4	-1.32 [-1.56, -1.08]	<0.001	0	0.600	⨁◯◯◯^2^ VERY LOW
NR	5	-1.98 [-3.59, -0.36]	0.020	98	<0.001	⨁◯◯◯^1,2^ VERY LOW
T ratio						
<0.714	4	-1.19 [-1.67, -0.70]	<0.001	40	0.170	⨁◯◯◯^1,2^ VERY LOW
0.714-0.825	2	-1.85 [-2.09, -1.60]	<0.001	99	<0.001	⨁◯◯◯^1,2^ VERY LOW
0.825-0.886	3	-1.35 [-1.58, -1.11]	<0.001	84	<0.001	⨁◯◯◯^1,2^ VERY LOW
<0.886	4	-0.74 [-0.88, -0.60]	<0.001	91	<0.001	⨁◯◯◯^,2^ VERY LOW
NR	7	-0.52 [-0.73, -0.31]	<0.001	87	<0.001	⨁◯◯◯^1,2^ VERY LOW

Abbreviations: WMD, Weighted Mean Difference; HOMA-IR, homeostasis model assessment of insulin resistance; T ratio, Total testosterone ratio; NR, no reported.

^1^Downgraded by 1 level for substantial heterogeneity (*I*2 statistic is greater than 40%)

^2^Downgraded by two levels for the limitations of the included studies and lower total sample size

**Table 4:** Univariate meta-regression analysis for the potential variables between studies

**Covariates**	**No. studies**	**Coefficient**	**Standard error**	***t***	***P***	**95% CI**
BMI	20	-0.794	0.575	-1.38	0.185	[-2.004,0.150]
Duration	20	-0.464	0.541	-0.86	0.402	[-1.600,0.673]
Dose	20	0.205	0.609	0.34	0.741	[-1.074,1.483]
HOMA-IR ratio	20	-0.243	0.171	-1.42	0.172	[-0.601,0.116]
T ratio	20	0.128	0.184	0.70	0.496	[-0.259,0.515]
Quality score	20	0.758	0.546	1.39	0.182	[-0.391,1.908]

**Figure 3:**
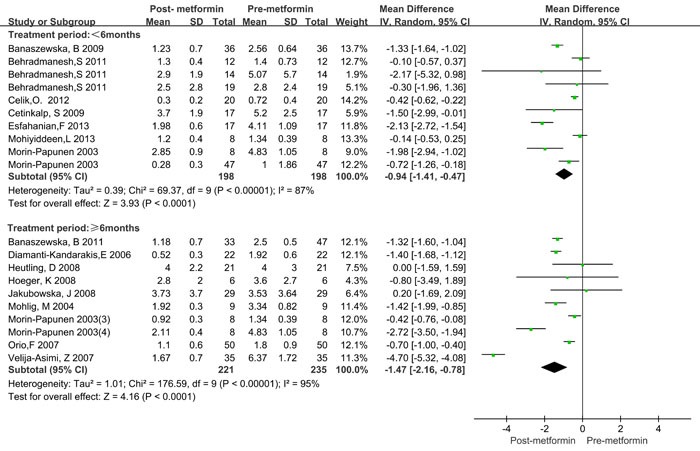
Forest plot for subgroup analysis stratified by treatment duration

**Figure 4:**
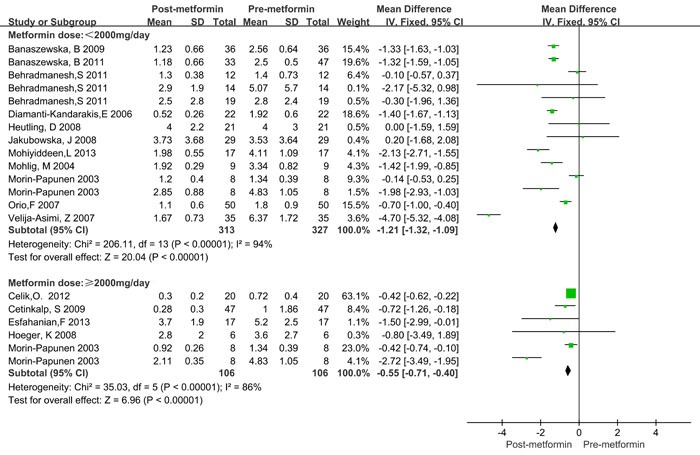
Forest plot for subgroup analysis stratified by metformin dose

Since significant heterogeneity was observed, a subgroup analysis was conducted. BMI was categorized as ≥ 25kg/m^2^ and < 25kg/m^2^_._ Treatment time was categorized as < 6 months and ≥ 6 months. The treatment dose of metformin was categorized as < 2000mg/day and ≥ 2000mg/day. The HOMA2-IR ratio and T ratio were categorized according to quartile intervals. As shown in Table 3, significantly decreased CRP levels were observed both in lean (BMI < 25 kg/m2) and obese (BMI > 25 kg/m2) patients, as well as in the treatment duration and dose subgroup. Similar results were also observed in different categories of HOMA2-IR ratio and total testosterone ratio values. Moreover, the CRP levels decreased more in obese patients (WMD = -1.48 mg/L, 95%CI: -2.16 to -0.80) than in lean patients (WMD = -0.79 mg/L, 95%CI: -1.28 to -0.30), and more in ≥ 6 months (WMD = -1.47 mg/L, 95%CI: -2.16 to -0.78) than in < 6 months (WMD = -1.48 mg/L, 95%CI: -1.41 to -0.47, Figure 3). However, treatment with metformin with ≥ 2000mg/day did not show any significant beneficial effects on CRP levels (WMD = -0.55 mg/L for ≥ 2000mg/day and WMD = -0.21 mg/L for < 2000mg/day, Figure 4). Significant heterogeneity was still observed in the subgroup analysis.

To further investigate which study characteristics of those mentioned above affected the WMD in CRP levels, we performed a meta-regression analysis. WMD was used as the dependent variable, and BMI was used at the baseline; treatment duration, daily dose, HOMA-IR ratio, and total testosterone ratio and sample size were entered as explanatory covariates. As shown in Table 3, BMI (20 studies, *P* = 0.185), treatment duration (20 studies, *P* = 0.402), daily dose (20 studies, *P* = 0.741), HOMA-IR ratio (17 studies, *P* = 0.172), and total testosterone ratio (20 studies, *P* = 0.496) were assessed independently. Since no regression coefficients of the covariates were significant at the level of 0.1, multivariate meta-regression was not carried out further.

### Sensitivity analysis

We first excluded studies one by one, and the pooled WMD was not significantly changed. We further excluded studies by Morin-Papunen *et al.* [22], Hoeger *et al.* [28], and Mohlig *et al.* [21] because these studies included small sample size (PCOS patients < 10), and the recalculated results did not significantly change (WMD = -1.23 mg/L, 95%CI:-1.75 to -0.71, *P* < 0.001). Using a Galbraith plot analysis, we excluded the outliers that might cause heterogeneity, the CRP levels still decreased significantly after treatment with metformin (WMD = -0.83 mg/L, 95%CI:-1.12 to -0.55); however, the heterogeneity also decreased significantly (*I*2 = 11% and *P*Q = 0.34, Supplementary Figure 1).

### Publication bias and quality of evidence

Begg's tests (*P* = 0.626, Supplementary Figure 2) and Egger’ tests (*P* = 0.278) showed that no significant publication bias was detected.

The quality of the evidence was shown in Table 3. Considering the included studies were all observational studies (before-after), evidence was downgraded for failure to adequately control confounding. Several comparisons had statistically significant heterogeneity, hence, evidence was downgraded for inconsistency. Evidence was also downgraded for the limitations of the included studies and lower total sample size. Therefore, the quality of the evidence for all outcomes should be rated as being very low.

## DISCUSSION

In this meta-analysis, we investigated whether metformin decreased CRP levels in women with PCOS. Our results showed that in women with PCOS, CRP levels significantly decrease after metformin treatment, especially in obese women with PCOS. Interestingly, we observed a significant time effect of metformin treatment on CRP levels in women with PCOS, but not with a dose relationship. However, the quality of evidence is very low.

Decreased levels of CRP in women with PCOS after metformin treatment may be related to PCOS’ biochemical characteristics, such as obesity and IR. Obesity is a major factor associated with elevated CRP in individuals with the metabolic syndrome [30], and CRP decreases after weight loss in obese subjects [31]. Harborne *et al*. found that weight loss is a feature of metformin therapy in obese women with PCOS, with a dose relationship [32]. Therefore, our study found that CRP levels decrease more in obese patients. There is a significant correlation at the baseline between CRP and insulin response [33], and high levels of CRP are associated with hyperinsulinemia [34]. Thus, with the improvement to IR in women with PCOS after metformin treatment, the elevated levels of CRP decrease.

Metformin can also reduce the levels of total testosterone in women with PCOS [35]. Indeed, in this study, decreased levels of total testosterone after metformin treatment were found in most of the included studies (T ratio < 1, Table 2). Previous studies have demonstrated an inverse relationship between total testosterone and CRP [36, 37]. However, we observed CRP levels decreased in different categories of T ratio. Interestingly, the decreased levels of CRP were also found in the subgroup with a HOMA-IR ratio ≥ 1. In addition, we did not observed a dose effect of metformin on CRP levels; in contrast, CRP levels decreased more in the subgroup with a dose of < 2000mg/day than that of ≥ 2000mg/day. These results suggest that decreased CRP levels are a result of multiple factors in women with PCOS.

PCOS is a life-time heterogeneous endocrinopathy, and long-term management of this frequent disorder must consider all the consequences of the syndrome, including T2DM and cardiovascular events [38, 39]. Kong *et al*. [40] found that metformin therapy could increase serum adiponectin concentrations and decrease serum leptin levels in women with PCOS. A systematic review also indicated that serum interleukin-6 levels of PCOS patients may be influenced by metformin [41]. CRP is a classical marker of inflammation that has be shown in persons with increased or sustained elevated CRP levels over a 6-year period has an increased risk of incident diabetes, coronary heart disease, ischemic stroke, heart failure, and mortality [42], moreover, the greatest hazard reduction in vascular events are observed in participants who achieved hs-CRP concentrations less than 1 mg/L in a prospective study [43]. On the basis of this meta-analysis and previous work, metformin is clearly a drug that is beneficial for chronic low-grade inflammation in women with PCOS. Therefore, metformin administration offers new possibilities for preventing CVD in women with PCOS, considering the important role of CRP in CVD.

This meta-analysis has several limitations. First, we noticed that all the sample sizes in the included studies were small (*n* < 50), with some even less than 10. This is probably the main reason for significant heterogeneity in this meta-analysis because significant heterogeneity still existed after the subgroup analysis, and the meta-regression analysis also could not determine the source of heterogeneity. The conflicting results in the subgroup analysis also may be caused by the small sample sizes. Second, since significant heterogeneity was observed in this study, a funnel plot of the data was not applied, as this may be incorrectly interpreted when studies are heterogeneous [44]. Third, some of the included studies had missing data for HOMA-IR, which suggests a certain degree of systemic bias in the association between the decreased levels of CRP and IR status. In addition, most of the included studies focused on Caucasian populations. Therefore, potential publication bias should be noted, which may influence the reliability of conclusions, although a Begg's funnel plot and an Egger's test showed no publication bias. Finally, the quality of evidence was very low, therefore, large studies, especially randomized trials are still needed.

Despite the above considerations, our study has some advantages. We assessed heterogeneity using various statistical methods. The pooled results were not significantly changed using a sensitivity analysis and a Galbraith plot analysis. In particular, the heterogeneity decreased significantly in the Galbraith plot analysis.

In summary, this study suggests that metformin has the potential effects on CRP levels in women with PCOS. However, considering the very low quality of evidence for the analysis, the effect of metformin on CRP levels are still very uncertain, and large-scale randomized-controlled study is needed to ascertain the long-term effects of metformin in PCOS.

## MATERIALS AND METHODS

This meta-analysis was strictly conducted according to the PRISMA 2009 statement (preferred reporting items for systematic reviews and meta-analyses), including the search strategy, selection criteria, data extraction, and data analysis [45](Supplementary Table 1).

### Search strategy

PubMed, Embase, Scopus and the Cochrane Library were systematically searched by two investigators (YC and ML) up to November 2016 to obtain eligible studies. The following search terms were used: *polycystic ovary disease*, *PCOD*, *polycystic ovary syndrome*, *PCOS*, *hyperandrogenism*, *CRP*, *C-reactive protein*, and *metformin* (S1 file) Articles in reference lists were also hand searched. Any divergence was resolved through consultation with a third reviewer.

### Inclusion and exclusion criteria

Inclusion criteria included (1) studies in which women with PCOS were treated with metformin, (2) studies with diagnostic criteria for PCOS, and (3) studies that reported the CRP means and standard deviation (SD) before and after treatment with metformin in PCOS women.

Exclusion criteria included (1) studies with no diagnostic criteria; (2) studies conducted on patients with other diseases or with a combination of other types of drugs; (3) studies without CRP levels (and where this information was not available from the contacted authors); and (4) review articles, letters, case reports, editorials, and conference abstracts.

### Data extraction

The following data were extracted by two investigators (YC and HD) from the included studies: the first author's name, publication year, country, total sample size, diagnostic criteria for PCOS, clinical variables (age and BMI), metformin information (dose and duration of metformin treatment), CRP means and SD (before and after metformin treatment), IR and total testosterone levels (before and after metformin treatment), and the measurement method for CRP. The investigators were instructed to try to contact the author to get the original data by email if the literature did not provide sufficient data. In cases of a dispute, the two investigators were obliged to check their data again, and any discrepancy was resolved by all authors through consultation.

### Quality assessment

The quality of the included studies in this meta-analysis was assessed according to the Newcastle-Ottawa Quality Assessment Scale (NOS) [46], with some of the criteria modified (Table 1). Studies with an NOS score ≤ 7 were considered “Low” quality studies. “High” quality studies were defined as having a quality score 8. The maximum NOS score was 10. We assessed the quality of the evidence using the GRADE approach (http://gdt.guidelinedevelopment.org)

### Statistical analysis

CRP and total testosterone levels in each study were extracted as mean difference ± SD. Homeostasis model assessment of insulin resistance (HOMA2-IR) values were used as measures of insulin sensitivity. The weighted mean differences (WMDs) and 95% confidence intervals (CIs) of the CRP levels were used as a measure of effect size, as the CRP levels in included studies were measured in the same units and the mean level difference was small across studies.

Heterogeneity was analyzed using Cochran's Q and Higgins's I-squared (*I*2) statistics. If *I*2 < 50% or *P*Q ≥ 0.1, there is no significant amount of heterogeneity between studies and therefore, a fixed-effects model was conducted using the Mantel-Haenszel method. Otherwise, a random-effects model (the DerSimonian and Laird method) was used. If significant heterogeneity was observed, a subsequent subgroup analysis was carried out according to BMI, metformin dose and duration, HOMA2-IR ratio, and total testosterone ratio (T ratio). Univariate meta-regression analyses were further performed to investigate the source of heterogeneity. Covariates with values of *P* < 0.05 were considered the main sources of heterogeneity; however, variables that were significant at the 0.1 level were entered into the multivariable. Galbraith plot analysis was used to find the outliers that might cause the heterogeneity.

Sensitivity analysis was performed by omitting the studies one by one and recalculating the pooled WMD. Begg's test and Egger's test were performed to evaluate the potential publication bias.

All *P* values were two-sided, and a *P*_value_ < 0.05 was considered statistically significant. A meta-analysis was conducted using Review Manager (version 5.3; Oxford, UK: the Cochrane Collaboration) and STATA software (version 12.0; Stata Corporation, College Station, Texas, USA).

## SUPPLEMENTARY MATERIALS FIGURES AND TABLES






