# Supplementary material for: Impact of metformin on C-reactive protein levels in women with polycystic ovary syndrome: a meta-analysis
Source: Oncotarget. 2017 Mar 8;8(21):35425–34. doi: 10.18632/oncotarget.16019 (PMC5471066; doi:10.18632/oncotarget.16019)
Supplement: Supplementary file 1 [file oncotarget-08-35425-s001.pdf]

Impact of metformin on C-reactive protein levels in women with polycystic ovary syndrome: a meta-analysis

Supplementary Material

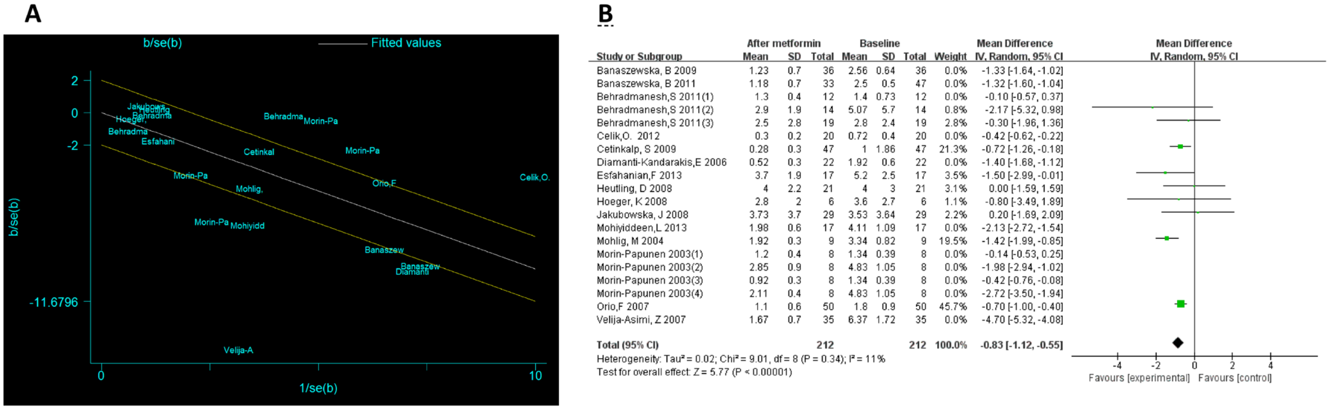

Supplementary Figure 1: Galbraith plot analysis (A) and forest plot for pooled analysis after excluded outlier.

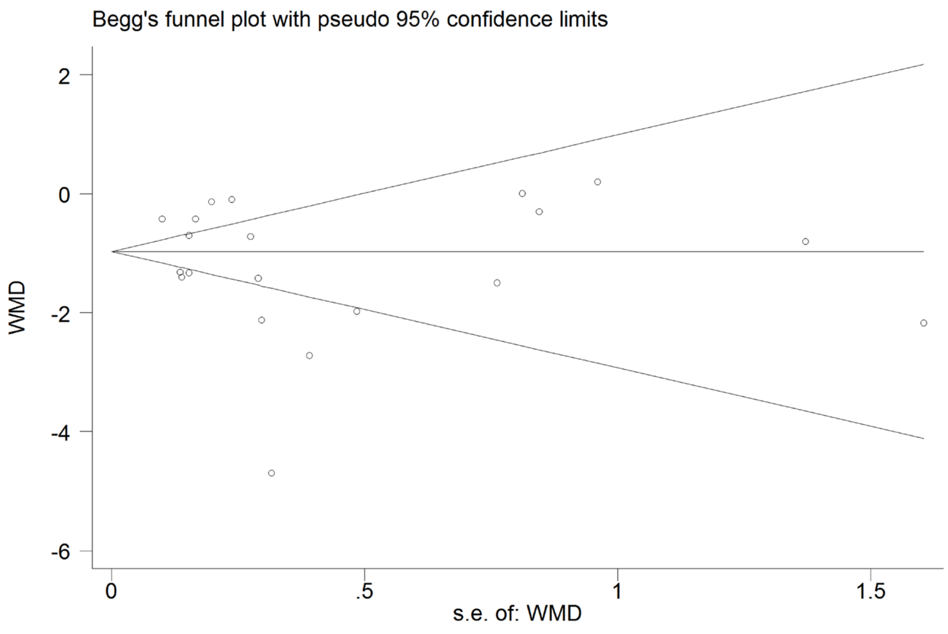

Supplementary Figure 2: Begg's funnel plot

SupplementaryFile 1: Search strategy and exclusion with reasons
Supplementary Table 1: PRISMA 2009 Checklist

For Supplementary Table 1 and Supplementary File 1 see in supplementary Information
