# Supplementary material for: Impact of metformin on C-reactive protein levels in women with polycystic ovary syndrome: a meta-analysis
Source: Oncotarget. 2017 Mar 8;8(21):35425–34. doi: 10.18632/oncotarget.16019 (PMC5471066; doi:10.18632/oncotarget.16019)
Supplement: Supplementary file 3 [file oncotarget-08-35425-s003.pdf]

Search strategy

**((metformin[Title/Abstract]) AND ((C-reactive protein[Title/Abstract]) OR CRP[Title/Abstract])) AND (((((PCOS[Title/Abstract]) OR hyperandrogenism[Title/Abstract]) OR polycystic ovary syndrome[Title/Abstract]) OR PCOD[Title/Abstract]) OR polycystic ovary disease[Title/Abstract])**

## Exclusion with reasons

- 7:meta-analysis[1],review[2-6],Posters[7]
- 2:animal experiment[8, 9]
- 2:not PCOS patients[10, 11]
- 8: no metformin treatment group [12-19]
- 27: CRP values were absent or did not represent mean±SD [20-46]


1. Sun, J., et al., *An investigation into the therapeutic effects of statins with metformin on polycystic ovary syndrome: A meta-analysis of randomised controlled trials.* BMJ Open, 2015. **5**(3).
2. Bhatia, V., *Insulin resistance in polycystic ovarian disease.* Southern Medical Journal, 2005. **98**(9): p. 903-912+923.
3. Dhindsa, G., et al., *Insulin resistance, insulin sensitization and inflammation in polycystic ovarian syndrome.* Journal of Postgraduate Medicine, 2004. **50**(2): p. 140-144.
4. Martin-Du Pan, R.C. and J. Ruiz, *Metabolic syndrome: Jumble syndrome of obese people or specific entity? Which treatment: Diet or polypill?* Revue Medicale Suisse, 2008. **4**(143): p. 366-373.
5. Vrbikova, J., *[Polycystic ovary syndrome].* Vnitr Lek, 2015. **61**(10): p. 886-95.
6. Ibañez, L., A. López-Bermejo, and F. De Zegher, *Treatment of PCOS in non-obese adolescents.* Hormone Research in Paediatrics, 2013. **80**: p. 24.
7. Tütüncü, L., S. Bodur, and O. Dundar, *Effects of 3 mg drospirenone/30 mg ethinyl estradiol alone or combined with metformin on metabolic risk factors in women with PCOS.* Human Reproduction, 2010. **25**: p. i302-i303.
8. Gozukara, I.O., et al., *Effect of colchicine on polycystic ovary syndrome: an experimental study.* Arch Gynecol Obstet, 2016. **293**(3): p. 675-80.
9. Pai, S.A. and A.S. Majumdar, *Protective effects of melatonin against metabolic and reproductive disturbances in polycystic ovary syndrome in rats.* The Journal of pharmacy and pharmacology, 2014. **66**(12): p. 1710-1721.
10. Ibáñez, L., et al., *Early metformin therapy (age 8-12 years) in girls with precocious pubarche to reduce hirsutism, androgen excess, and oligomenorrhea in adolescence.* Journal of Clinical Endocrinology and Metabolism, 2011. **96**(8): p. E1262-E1267.

11. Ibañez, L., et al., *Towards early metformin therapy (age 8-12 yr) to prevent Polycystic Ovary Syndrome (PCOS) in adolescence.* Hormone Research in Paediatrics, 2011. **76**: p. 38.
12. Dawson, A.J., et al., *Exenatide reduces body weight but not hyperandrogenemia in overweight women with polycystic ovary syndrome.* Endocrine Reviews, 2011. **32**(3).
13. Flannery, C.A., et al., *Metabolic testing in adolescents with polycystic ovary syndrome.* Diabetes, 2010.
14. Raja-Khan, N., et al., *High-dose vitamin D fails to improve insulin sensitivity in women with polycystic ovary syndrome.* Endocrine Reviews, 2012. **33**(3).
15. Ghazeeri, G., et al., *Inadequacy of initiating rosuvastatin then metformin on biochemical profile of polycystic ovarian syndrome patients.* Journal of Endocrinological Investigation, 2015. **38**(6): p. 643-651.
16. Ibáñez, L., C. Valls, and F. de Zegher, *Discontinuous low-dose flutamide-metformin plus an oral or a transdermal contraceptive in patients with hyperinsulinaemic hyperandrogenism: Normalizing effects on CRP, TNF-α and the neutrophil/lymphocyte ratio.* Human Reproduction, 2006. **21**(2): p. 451-456.
17. Ilie, I.R., et al., *Ethinylestradiol30μg-drospirenone and metformin: Could this combination improve endothelial dysfunction in polycystic ovary syndrome?* BMC Endocrine Disorders, 2012. **12**.
18. Sathyapalan, T., et al., *Atorvastatin reduces malondialdehyde concentrations in patients with polycystic ovary syndrome.* Journal of Clinical Endocrinology and Metabolism, 2012. **97**(11): p. 3951-3955.
19. Ajjan, R., et al., *Clot structure and fibrinolysis in individuals with polycystic ovary syndrome: The effects of metformin, orlistat and pioglitazone treatment.* Endocrine Abstracts, 2009. **19**: p. P282.
20. Agarwal, N., et al., *Metformin improves arterial stiffness and endothelial function in young women with PCOS: A randomised, crossover trial.* Endocrine Abstracts, 2009. **19**: p. OC24.
21. Agarwal, N., et al., *Metformin reduces arterial stiffness and improves endothelial function in young women with polycystic ovary syndrome: A randomized, placebo-controlled, crossover trial.* Journal of Clinical Endocrinology and Metabolism, 2010. **95**(2): p. 722-730.
22. Christakou, C., et al., *The benefit-to-risk ratio of common treatments in PCOS: Effect of oral contraceptives versus metformin on atherogenic markers.* Hormones, 2014. **13**(4): p. 488-497.
23. Christakou, C., et al., *Effect of pharmaceutical intervention on serum advanced glycated end products levels in women with polycystic ovary syndrome.* Endocrine Reviews, 2013. **34**(3).
24. Dardzinska, J.A., et al., *Effects of metformin or an oral contraceptive containing cyproterone acetate on serum c-reactive protein, interleukin-6 and soluble vascular cell adhesion molecule-1 concentrations in women with polycystic ovary syndrome.* Exp Clin Endocrinol Diabetes, 2014. **122**(2): p. 118-25.
25. Glintborg, D., et al., *Increased thrombin generation in women with polycystic ovary syndrome: A pilot study on the effect of metformin and oral contraceptives.* Metabolism: Clinical and Experimental, 2015. **64**(10): p. 1272-1278.
26. Jensterle, M., et al., *Improvement of endothelial function with metformin and rosiglitazone treatment in women with polycystic ovary syndrome.* European Journal of Endocrinology, 2008. **159**(4): p. 399-406.

27. Kilic, S., et al., *Inflammatory-metabolic parameters in obese and nonobese normoandrogenemic polycystic ovary syndrome during metformin and oral contraceptive treatment.* Molecular Human Reproduction, 2009. **24**: p. i183.
28. Kilic, S., et al., *Inflammatory-metabolic parameters in obese and nonobese normoandrogenemic polycystic ovary syndrome during metformin and oral contraceptive treatment.* Gynecological Endocrinology, 2011. **27**(9): p. 622-629.
29. Navali, N., et al., *Therapeutic effects of biguanide vs. statin in polycystic ovary syndrome: A randomized clinical trial.* Pakistan Journal of Biological Sciences, 2011. **14**(11): p. 658-663.
30. Preiss, D., et al., *The effects of 8 months of metformin on circulating GGT and ALT levels in obese women with polycystic ovarian syndrome.* International Journal of Clinical Practice, 2008. **62**(9): p. 1337-1343.
31. Tan, B.K., et al., *Metformin treatment may increase omentin-1 levels in women with polycystic ovary syndrome.* Diabetes, 2010. **59**(12): p. 3023-3031.
32. Tan, B.K., et al., *Metformin increases the novel adipokine cartonectin/ctrp3 in women with polycystic ovary syndrome.* Journal of Clinical Endocrinology and Metabolism, 2013. **98**(12): p. E1891-E1900.
33. Tan, B.K., et al., *Circulatory changes of the novel adipokine adipolin/CTRP12 in response to metformin treatment and an oral glucose challenge in humans.* Clinical Endocrinology, 2014. **81**(6): p. 841-846.
34. Teede, H.J., et al., *Endothelial function and insulin resistance in polycystic ovary syndrome: the effects of medical therapy.* Fertility and Sterility, 2010. **93**(1): p. 184-191.
35. Tsilchorozidou, T., V. Mohamed-Ali, and G.S. Conway, *Determinants of interleukin-6 and C-reactive protein vary in polycystic ovary syndrome, as do effects of short- and long-term metformin therapy.* Hormone Research, 2009. **71**(3): p. 148-154.
36. Vanky, E., et al., *Beneficial effect of metformin on pregnancy outcome in women with polycystic ovary syndrome is not associated with major changes in C-reactive protein levels or indices of coagulation.* Fertility and Sterility, 2006. **85**(3): p. 770-774.
37. Velija-Asimi, Z., *Evaluation of endocrine changes in women with the polycystic ovary syndrome during metformin treatment.* Bosn J Basic Med Sci, 2013. **13**(3): p. 180-5.
38. Aghamohammadzadeh, N., et al., *Comparison of metformin and cyproteroneestrodiol compound effect on hs c-reactive protein and serum androgen levels in patients with poly cystic ovary syndrome.* Pakistan Journal of Medical Sciences, 2010. **26**(2): p. 347-351.
39. Al-Youzbaki, W.B. and R.G. Abdullah, *C-reactive protein and lipid profile in patients with polycystic ovary syndrome treated by metformin.* Pakistan Journal of Medical Sciences, 2013. **29**(2).
40. Fruzzetti, F., et al., *Adolescents with classical polycystic ovary syndrome have alterations in the surrogate markers of cardiovascular disease but not in the endothelial function. The possible benefits of metformin.* J Pediatr Adolesc Gynecol, 2016.
41. Gharakhani, M., N. Neghab, and M. Farimani, *Is reducing ovarian volume in polycystic ovarian syndrome patients after administration of metformin associated with improving cardiovascular risk factors?* International Journal of Fertility and Sterility, 2011. **5**(2): p. 90-95.

42. Kjotrod, S.B., et al., *C-reactive protein levels are unaffected by metformin during pretreatment and an IVF cycle in women with polycystic ovary syndrome.* Fertil Steril, 2008. **89**(3): p. 635-41.
43. Moran, L.J., et al., *Novel inflammatory markers in overweight women with and without polycystic ovary syndrome and following pharmacological intervention.* Journal of Endocrinological Investigation, 2010. **33**(4): p. 258-265.
44. Mehrabian, F., et al., *Comparison of the effects of metformin, flutamide plus oral contraceptives, and simvastatin on the metabolic consequences of polycystic ovary syndrome.* Journal of Research in Medical Sciences, 2016. **21**(1).
45. Duleba, A., et al., *Metformin, but not simvastatin, reduces serum levels of advanced glycation end products in women with PCOS: Results of a randomized trial.* Molecular Human Reproduction, 2009. **24**: p. i181.
46. Farimaini, M., et al., *Metformin decreased ovarian volume and effect on metabolic and hormonal in PCOS.* Iranian Journal of Reproductive Medicine, 2010. **8**: p. 8.
